# Fluid Platelet-Rich Fibrin (PRF) Versus Platelet-Rich Plasma (PRP) in the Treatment of Atrophic Acne Scars: A Comparative Study

**DOI:** 10.1007/s00403-022-02511-3

**Published:** 2022-12-15

**Authors:** Nagwa Ali Fahmy Diab, Al-shimaa M. Ibrahim, Aya Mohamed Abdallah

**Affiliations:** Dermatology, Venereology and Andrology, Faculty of Medicine, Zagazig University, Zagazig, Egypt

**Keywords:** Scar, PRF, Wound

## Abstract

Platelet-rich fibrin (PRF), a second-generation platelet concentrate, was developed for the purpose of overcoming the limitations of Platelet-rich plasma (PRP). PRF can produce a higher cumulative release of growth factors than PRP. Also, this release is slow and prolonged, making it ideal for tissue regeneration and growth stimulation. This study was conducted to evaluate the efficacy of fluid PRF either alone or combined with needling versus PRP in the treatment of atrophic acne scars. A comparative study including 30 patients with atrophic acne scars who were divided into two equal groups. Group I included 15 patients in which the left side of the face was treated with intradermal injection of PRP while the right side was treated with combined needling with PRP. Group II included15 patients in which the left side of the face was treated with intradermal injection of fluid PRF while the right side was treated with combined needling with fluid PRF**.** All patients received four sessions with 3 weeks interval. The acne scars significantly improved in both sides of face in both groups. According to quartile grading scale and patient satisfaction; the therapeutic response was significantly higher in PRF group than PRP either alone or combined with needling. The combination with needling increases efficacy of PRF and PRP. Fluid PRF is highly effective, safe and simple procedure that can be used instead of PRP in the treatment of acne scars.

## Introduction

Platelet-rich plasma (PRP), the first-generation platelet concentrate, has been used in the treatment of various diseases in different specialties like dermatology, orthopedics, and dentistry. PRP contains high level of growth factors which have marked growth potential and induce faster healing [1], so it becomes popular in regenerative medicine due to the convenient availability of growth factors that only needs drawing blood. However, different limitations of PRP were reported. The first one was the usage of external anticoagulant. In addition, the release of growth factors is closely linked with the clotting mechanism and the addition of anticoagulants could affect this release [2].

Other limitation of PRP is the rapid release of growth factors of PRP on activation. Approximately 95% of these factors are released shortly after activation with calcium chloride. These limitations led to the need of development of platelet concentrates without anticoagulant [3].

Platelet-rich fibrin (PRF), the second-generation platelet concentrate, was developed for the purpose of removing anticoagulants for fear of hypersensitivity reaction and for better release of growth factors. A rapid and short centrifugation procedure is needed for separation of blood layers before clotting. A fibrin matrix is formed in the platelet-rich layer entrapping platelets and leukocytes in it. This matrix makes the release of growth factors slow and prolonged comparing with PRP [4].

There are different preparations of PRF such as; Leukocyte-rich PRF (l-PRF), the platelets and WBCs are entrapped in the fibrin clot.The second preparation is Advanced PRF (A-PRF) that is associated with higher release of growth factors [5, 6]. Injectable-PRF (I-PRF) or fluid PRF has been reported to have higher levels of platelets and WBC than L-PRF and A-PRF. It remains in a liquid state for 15–20 min before coagulation, during which the I-PRF can be injected into the face, scalp or mixed with bone grafting materials [7].

Fluid PRF was investigated histologically; leukocytes (mostly lymphocytes) and platelets were found to be dispersed uniformly throughout the tested specimen, in contrast to PRF clots, where cells were distributed unevenly. The three-dimensional fibrin created in fluid PRF, together with growth factors; provide a controlled release system of growth factors throughout the healing process [8, 9].

The migration of fibroblasts is significantly more in fluid PRF than PRP. Fluid PRF is associated with significantly more cell proliferation and more elevation in fibronectin mRNA, collagen 1, and TGF-beta levels leading to greater induction of collagen synthesis [10].

The treatment of acne scar by skin micro-needling combined with PRP is more effective than skin micro-needling alone. The growth factors induced by both treatment modalities have a synergistic action which improves and accelerates wound healing [11].This study was conducted to evaluate the efficacy and safety of fluid PRF versus PRP either alone or combined with needling in treatment of post-acne atrophic scars.

## Patients and Methods

Thirty adult patients presented by atrophic acne scars of different severities were collected from the outpatient clinic of Dermatology at Z.U. Hospital after their acceptance to participate in this study. This study was approved by Z.U. Institutional Review Board (Z.U.IRB) IRB#: 5602-17-2-2020. Patients presented with history of keloid formation, immunosuppression, bleeding disorders, thrombocytopenia, Platelet dysfunction, patients with active skin or systemic disease and pregnancy were excluded from the study.

All patients were subjected to thorough history taking and dermatological examination to assess the type of scars, icepick, boxcar or rolling type, and the scar severity according to Goodman and Baron's qualitative global scarring grading system [12]. All patients were assigned randomly to two groups according to computer generated random list.

## Methods

### Preparation of PRP

10 ml of blood was drawn from every participant. Firstly, PRP and platelet-poor plasma (PPP) were separated from the RBC portion by centrifugation whole blood mixed with anticoagulant (EDTA) at 900 rpm for 5 min, and then second centrifugation was done at 2000 rpm for 15 min to separate PRP from platelet-poor plasma. About 2 mL of PRP was collected and 10% CaCl_2_ were added to PRP to be activated.

### Preparation of Fluid PRF

PRF was produced by single spin centrifugation of 10 ml of venous blood collected in plain glass tube without anticoagulant at 700 rpm for 3 min. The upper layer, yellow to orange colored fluid, was collected as fluid PRF. Approximately, 1 ml Fluid PRF can be separated from 10 ml blood.

### Procedure

45 min before the session, local anesthetic cream containing mixture of lidocaine and prilocaine was applied to the whole face. The whole face was sterilized by alcohol before starting the treatment.

- Left side of face was treated by intradermal injection of PRP (group1) or fluid PRF in (group 2). 0.1 mL of PRP or fluid PRF was injected intradermally into the atrophic scars with 1.5 to 2 cm interval using insulin syringe followed by gentle massaging of the treated area.
- For the treatment of right side of face in both groups: we used Derma electric-pen, (220v) and needle cartridge with 12 needles (Auto-Stamp Motorized Meso Machine, My-M, 500 mA, and 147*30). Needle length was adjusted at 2.5 mm and speed level 4 (blue color). PRP or fluid PRF were applied topically over the areas affected by acne scars followed by needling by moving dermapen in the four directions, vertically, horizontally, diagonally right and left, over the affected areas without pressing.
- The treatment started immediately after separation of PRF to avoid clot formation. All patients were instructed to apply topical antibiotic and sunscreen after each session. Any side effects were recorded every session.

All patients received four treatment sessions with 3 weeks interval followed by one month follow-up period.

### Assessment of the Therapeutic Response

The therapeutic response was assessed by

1. Goodman and Baron's global scarring grading system (GSGS): by comparing its values before the start of treatment and 4 weeks after the last session.
2. Quartile grading scale: the improvement was classified into: excellent if improvement > 75%; very good improvement 50–74%; good 25–49% and poor improvement < 25%.
3. Patient’s satisfaction: the patients assessed their degree of improvement as poor, good, very good and excellent. All patients were also asked to rate their pain on a scale of 0 to 10. 0 means no pain and 10 means the worst pain.

### Statistical Analysis

All data were collected, tabulated and statistically analyzed using (IBM SPSS Statistics for Windows, Version 23.0. Armonk, NY: IBM Corp.2015).

## Results

**Fig. 2:**
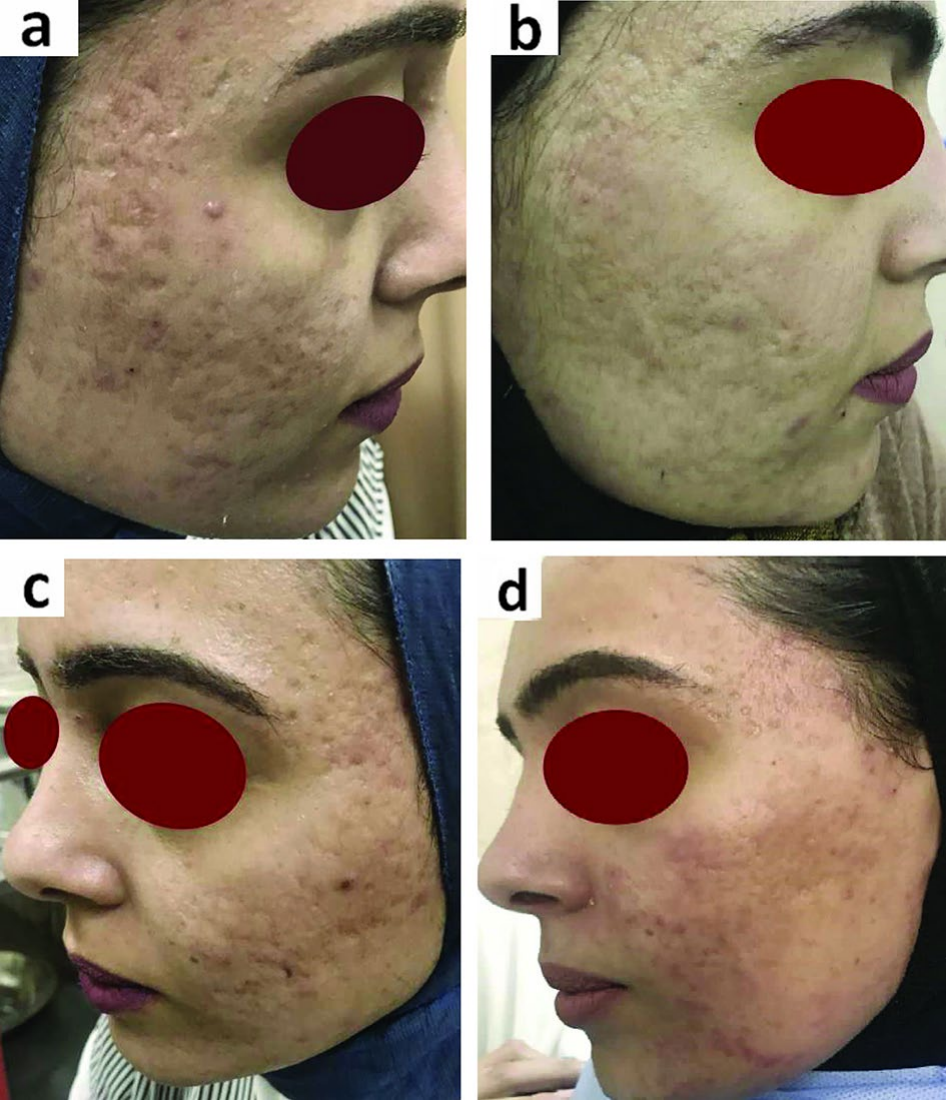
22 years old female patient had atrophic acne scar showed excellent improvement after treatment with PRF. Right side treated by combined fluid PRF with needling (**a**, before treatment), (**b**, after treatment). Left side of face treated by intradermal injection of PRF only (**c**, before treatment) (**d**, after treatment)

**Fig. 3:**
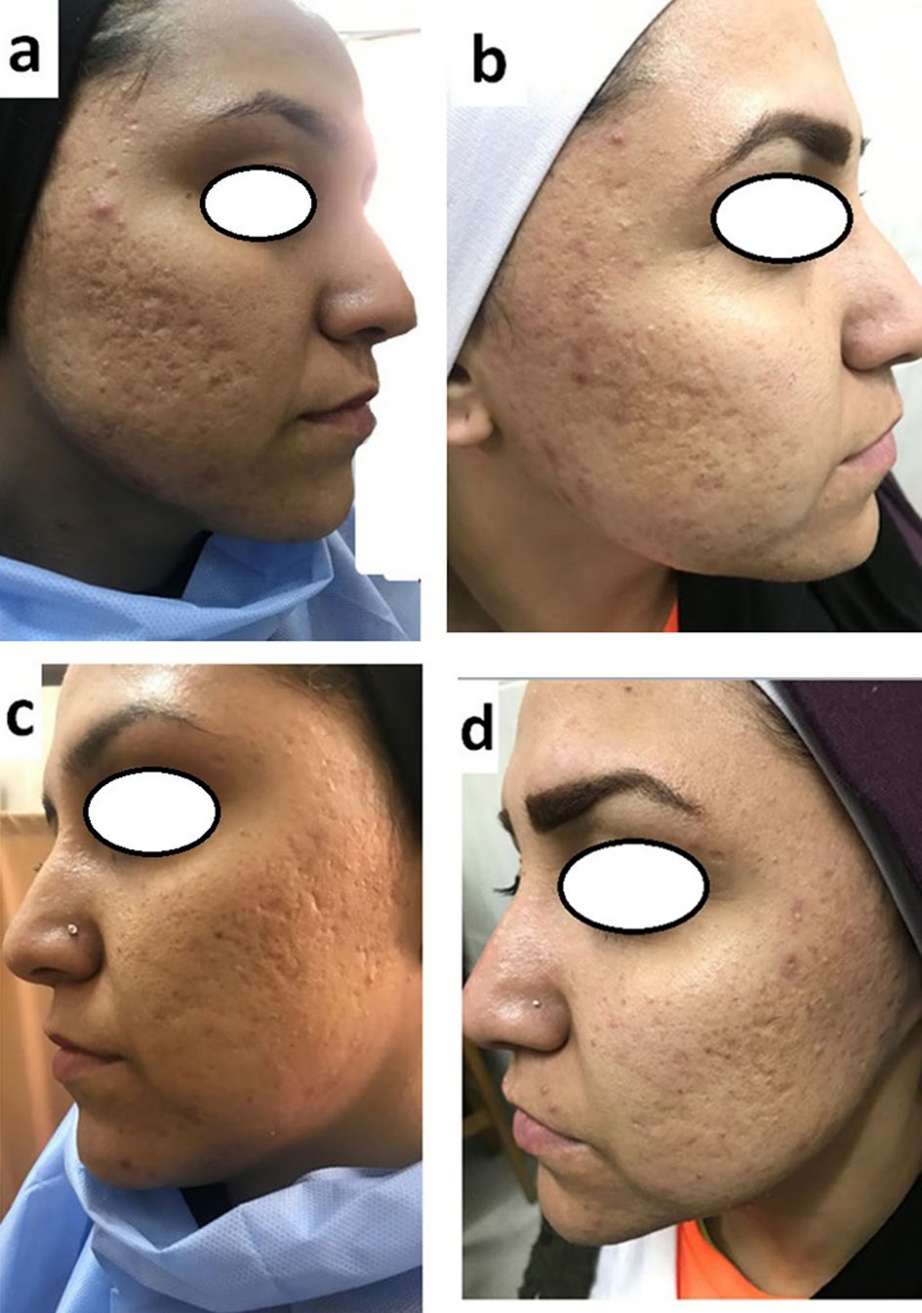
25 years old female patient had atrophic acne scar showed good improvement after treatment with PRP. Right side treated by combined PRP with needling (**a**, before treatment), (**b**, after treatment). Left side of face treated by intradermal injection of PRP only (**c**, before treatment) (**d**, after treatment)

- Before treatment there was no significant difference in acne scar severity in both sides of face of both groups according to GSGS.
- *Regarding PRP Group* It included 13 female and 2 males their age ranged from (18–33) years (mean age 24.53 ± 4.81 years). 93.3% of patients had positive family history of post-acne scar. Most of patients belonged to skin type IV (60%). Ice pick scar was the most common type of scars (73.3%) followed by rolling scar (66.7%). After treatment the severity of acne scar significantly improved in both sides of the face. The improvement was significantly higher in the right side treated by combined PRP and needling than left side treated by PRP alone according to quartile grading scale and patient satisfaction.
- *PRF Group* included 11 female and 4 males their ages ranged from (22–38) years (mean age 26.67 ± 4.76 years). 66.7% of patients had positive family history of post-acne scar. Most of patients belonged to skin type IV (73.3%). Rolling scar was the commonest scar (86.7%) followed by Icepick scar (46.7%). After treatment the severity of acne scar significantly improved in both sides of the face. The improvement was significantly higher in the right side treated by combined PRF and needling than left side treated by PRF alone according to quartile grading scale and GSGS (Table 1).
- *Fluid PRF versus PRP *According to the quartile grading scale and patient satisfaction; the improvement in PRF group either alone or combined with needling was significantly higher than PRP group. The severity of scar as assessed by GSGS showed more improvement in PRF than PRP, however, the difference between side treated by PRF alone and that treated by PRP alone was not statistically significant (Figs 1,2,3)  (Table 2).
  **Fig. 1:**
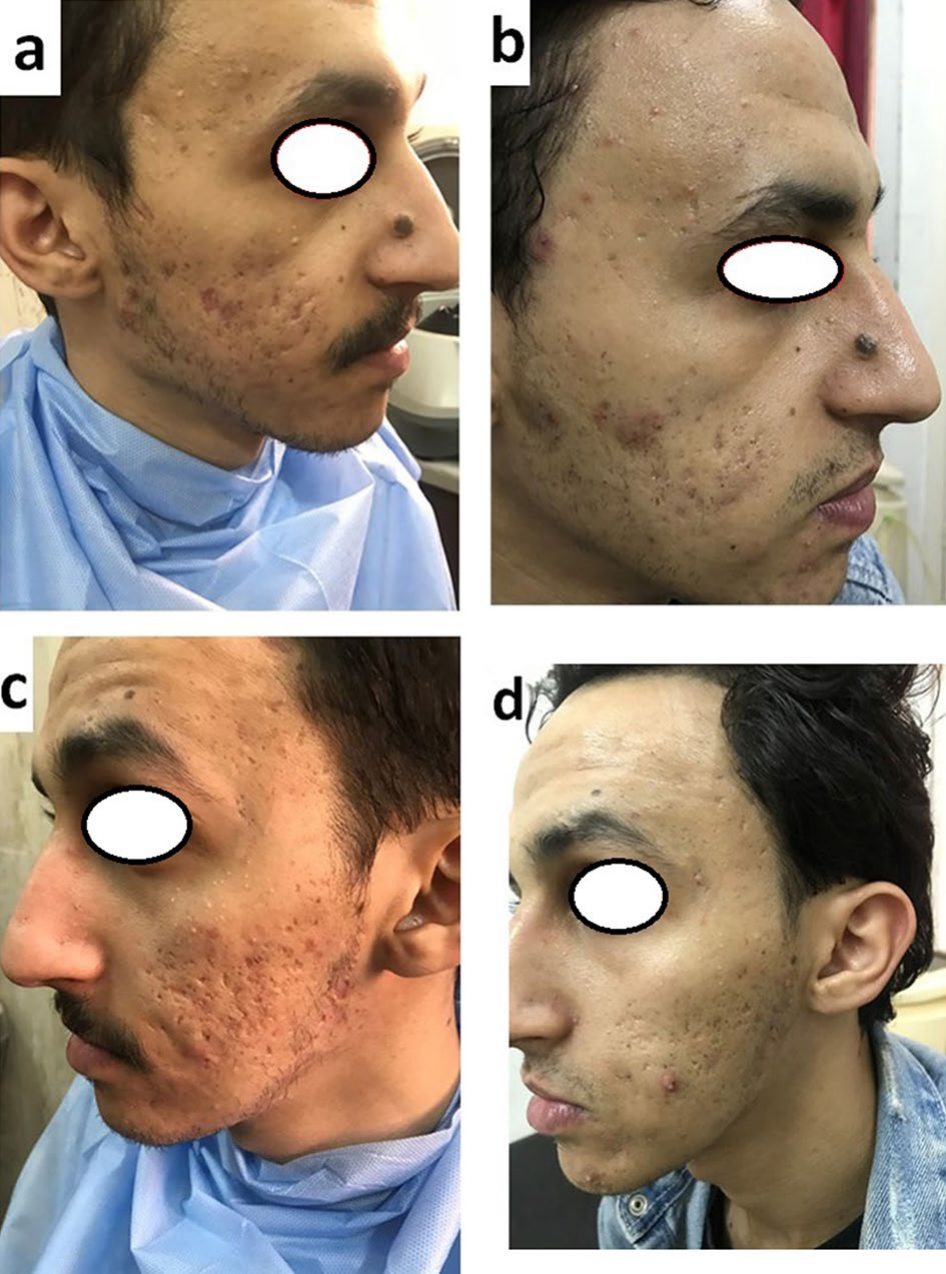
30 years old male patient had atrophic acne scar showed excellent improvement after treatment with PRF. Right side treated by combined fluid PRF with needling (**a**, before treatment), (**b**, after treatment). Left side of face treated by intradermal injection of PRF only (**c**, before treatment) (**d**, after treatment)

**Table 1:** Therapeutic response of the side treated by intradermal injection of PRP and PRF versus side treated by combined treatment of PRP or PRF with needling

Variable	Group I		*P*	Group II		*P*
	PRP Alone left side (*n* = 15)	PRP + micro-needling right side (*n* = 15)		PRF alone left side (*n* = 15)	PRF + micro-needling right side (*n* = 15)	
GSGS score Before treatment						
Median	10	10	**0.618**	12	12	**0.671**
Range	2-27	3-27		5-28	5-28	
*GSGS score*						
After treatment						
Median	8	6	**0.431**	6	4	0.02*
Range	2-18	2-15		3-12	2-11	
* P* value	0.003*	0.03*		0.003*	0.004*	
Response						
Poor	5 (33.3%)	2 (13.3%)	0.003*	2 (13.3%)	1 (6.7%)	0.001*
Good	6 (40%)	5 (33.3%)		6 (40%)	2 (13.3%)	
Very good	3 (20%)	5 (33.3%)		5 (33.3%)	5 (33.3%)	
Excellent	1 (6.7%)	3 (20%)		2 (13.3%)	7 (46.7%)	
Satisfaction						
Poor	7 (46.7%)	0 (0%)	0.01*	1 (6.7%)	0 (0%)	0.19**
Good	6 (40%)	7 (46.7%)		5 (33.3%)	1 (6.7%)	
Very good	1 (6.7%)	5 (33.3%)		4 (26.7%)	6 (40%)	
Excellent	1 (6.7%)	3 (20%)		5 (33.3%)	8 (53.3%)	

Data represented as median and range

Test: Sign test, Wilcoxon Rank Sum test

^*^Significant difference (*p* < 0.05)

^**^Nonsignificant difference (*p* > 0.05)

**Table 2:** Therapeutic response of PRP versus PRF according to GSGS, quartile grading scale and patient satisfaction

Variable	PRP Alone left side Group I (*n* = 15)	PRF alone left side Group II (*n* = 15)	*P*	PRP + micro-needling right side Group I (*n* = 15)	PRF + micro-needling right side Group II (*n* = 15)	*P*
GSGS before treatment						
Median	10	12	**0.302**	10	12	**0.33**
Range	2-27	5-28		3-27	5-28	
GSGS after treatment						
Median	8	6	**0.339**	6	4	0.02*
Range	2-18	3-12		2-15	2-11	
*P* value	0.003*	0.003*		0.03*	0.004*	
Response						
Poor	5 (33.3%)	2 (13.3%)	0.008*	2 (13.3%)	1 (6.7%)	0.003*
Good	6 (40%)	6 (40%)		5 (33.3%)	2 (13.3%)	
Very good	3 (20%)	5 (33.3%)		5 (33.3%)	5 (33.3%)	
Excellent	1 (6.7%)	2 (13.3%)		3 (20%)	7 (46.7%)	
Satisfaction						
Poor	7 (46.7%)	1 (6.7%)	0.02*	0 (0%)	0 (0%)	
Good	6 (40%)	5 (33.3%)		7 (46.7%)	1 (6.7%)	0.03*
Very good	1 (6.7%)	4 (26.7%)		5 (33.3%)	6 (40%)	
Excellent	1 (6.7%)	5 (33.3%)		3 (20%)	8 (53.3%)	

Data represented as number and percentage

Test: Sign test, Wilcoxon Rank Sum test

^*^Significant difference (*p* < 0.05)

The side effects in both groups were mild and tolerated with no significant difference between all groups. Facial skin appeared more erythematous and edematous in the side treated by combined treatment, but in all cases redness and edema disappeared with in 2–3 days after the sessions. Post-inflammatory hyperpigmentation was observed in 20% of sides treated by combined needling with PRP. Secondary bacterial infection occurred after first session in one patient of PRF group who improved after treatment by systemic antibiotic. Pain score was higher with combined treatment; however, the difference between both sides of face was not statistically significant and it was well tolerated by all patients (*p* > 0.05).

There was no significant relationship between therapeutic response and the age, sex, skin type and type of scars in both groups (*p* > 0.05).

## Discussion

Recently, PRF has been introduced in the treatment of different diseases to overcome the limitations of PRP. In PRF there is no need of anticoagulant, in addition to the gradual and extended release of growth factors by platelets and leukocytes entrapped within the fibrin matrix in PRF [6, 13, 14].

The term PRF is used for the solid PRF (Advanced PRF, and leukocyte PRF) and the term liquid or fluid PRF for Injectable PRF and Concentrate PRF. Leukocytes and platelets were found to be distributed uniformly throughout the histologically analyzed specimen of fluid PRF unlike in solid PRF, where the cells were unevenly distributed. The three-dimensional fibrin presents in fluid PRF forms a controlled release system of growth factors. This property makes fluid PRF a good alternative to PRF clot especially in the treatment of large surface areas [8–10].

Skin needling acts by creating micro-skin injuries to stimulate collagen remodeling and enhance new collagen and elastin formation, associated with improvement in the vascularization in the upper dermis. This effect leads to reduction in fine wrinkles, scarring with improvement in the skin laxity [15, 16].

Skin needling has been used in the treatment of acne scar either alone or combined with other treatments like PRP. The autologous growth factors in PRP and PRF work in synergism with growth factors created by skin needling to improve the collagen remodeling and wound healing. It was found that the growth factors in PRP were released almost totally in first 15 min of injection, requiring more number of sessions at frequent intervals [14, 16–18].

This study was conducted to evaluate the efficacy and safety of fluid PRF either alone or combined with needling versus PRP in treatment of atrophic post-acne scars.

All patients of both groups showed significant improvement in acne scars in both sides of face. The improvement was better in the side treated by combined needling with PRP or PRF than side treated by PRP or PRF alone. According to the quartile grading scale and patient satisfaction; the improvement in PRF group either alone or combined with needling was significantly higher than PRP group. We noticed more improvement in skin texture and skin laxity in PRF group compared to PRP group. The improvement was noticed earlier in PRF group than PRP group especially in side treated by intradermal injection of fluid PRF that had filling and lifting effect which was remarkable from the second day of injection and maintained up to 2 weeks after injection. This effect may explain the earlier response in PRF group by raising the scar deficit before the effect of stimulating growth factors to take place.

As regard side effects; the difference between both groups was not significant. Facial skin appeared more erythematous and edematous in the side treated by combined treatment, but in all cases redness and edema disappeared with in 2–3 days after the sessions. Similar side effects were reported in previous studies [17–20]. Post-inflammatory hyperpigmentation was observed in 20% of sides treated by combined needling with PRP. In this study, secondary bacterial infection occurred after first session in one patient of PRF group who improved after treatment by systemic antibiotic. Pain was well tolerated in all patients in our study.

In accordance with our results different study had reported improvement in acne scar by PRP [16–20].On the other hand, up to our knowledge there is no controlled studies that evaluate the efficacy of fluid PRF in the treatment of acne scar.

PRF has been used in the treatment of different diseases in dentistry, and orthopedics. Wang et al. [21] approved the greater regenerative potential of fluid PRF on cultivated human skin fibroblasts when compared with PRP. Shashank et al. [22] have used fluid PRF in treatment of androgenic alopecia, facial rejuvenation, temporary correction of facial skin folds and healing of wounds and reported promising results suggesting that fluid PRF can be an alternative to PRP.

This study showed promising findings regarding the efficacy and safety of fluid PRF, a simple, safe, rapid and cost-effective procedure, in the treatment of atrophic acne scar; however, there is still a need for further controlled trials on PRF either alone or combined with different treatment modalities to confirm the ability of PRF to substitute PRP in the treatment of different skin diseases.

## References

[1] Ghanaati S, Booms P, Orlowska A, Kubesch A, Lorenz J, Rutkowski J, Landes C, Sader R, Kirkpatrick CJ, Choukroun J (2014). Advanced platelet-rich fibrin: a new concept for cell-based tissue engineering by means of inflammatory cells. J Oral Implantol.

[2] Fujioka-Kobayashi M, Miron RJ, Hernandez M, Kandalam U, Zhang Y, Choukroun J (2017). Optimized platelet-rich fibrin with the low-speed concept: growth factor release, biocompatibility, and cellular response. J Periodontol.

[3] Miron RJ, Fujioka-Kobayashi M, Hernandez M, Kandalam U, Zhang Y, Ghanaati S, Choukroun J (2017). Injectable platelet rich fibrin (i-PRF): opportunities in regenerative dentistry?. Clin Oral Invest.

[4] Ehrenfest DM, Rasmusson L, Albrektsson T (2009). Classification of platelet concentrates: from pure platelet-rich plasma (P-PRP) to leucocyte-and platelet-rich fibrin (L-PRF). Trends Biotechnol.

[5] Fujioka-Kobayashi M, Katagiri H, Kono M, Schaller B, Zhang Y, Sculean A, Miron RJ (2020). Improved growth factor delivery and cellular activity using concentrated platelet-rich fibrin (C-PRF) when compared with traditional injectable (i-PRF) protocols. Clin Oral Invest.

[6] Kobayashi E, Flückiger L, Fujioka-Kobayashi M, Sawada K, Sculean A, Schaller B, Miron RJ (2016). Comparative release of growth factors from PRP, PRF, and advanced-PRF. Clin Oral Invest.

[7] Miron RJ, Chai J, Zhang P, Li Y, Wang Y, Mourão CF, Sculean A, Fujioka Kobayashi M, Zhang Y (2020). A novel method for harvesting concentrated platelet-rich fibrin (C-PRF) with a 10-fold increase in platelet and leukocyte yields. Clin Oral Invest.

[8] Thanasrisuebwong P, Surarit R, Bencharit S, Ruangsawasdi N (2019). Influence of fractionation methods on physical and biological properties of injectable platelet-rich fibrin: an exploratory study. Int J Mol Sci.

[9] Kobayashi M, Kawase T, Horimizu M, Okuda K, Wolff LF, Yoshie H (2012). A proposed protocol for the standardized preparation of PRF membranes for clinical use. Biologicals.

[10] de Almeida VH, de Araujo RF, Vasconcelos RC, Garcia VB, de Souza LB, de Araujo AA (2018). Histological preparation technique of blood derivative injectable platelet-rich fibrin (I-Prf) for microscopic analyzes. J Cytol Histol.

[11] Fabbrocini G, Annunziata MC, D'arco V, De Vita V, Lodi G, Mauriello MC, Pastore F, Monfrecola G (2010). Acne scars: pathogenesis, classification and treatment. Dermatol Res Pract.

[12] Goodman GJ, Baron JA (2006). Postacne scarring: a qualitative global scarring grading system. Dermatol Surg.

[13] Dohan Ehrenfest DM, Del Corso M, Diss A, Mouhyi J, Charrier JB (2010). Three-dimensional architecture and cell composition of a Choukroun's platelet-rich fibrin clot and membrane. J Periodontol.

[14] Dashore S, Chouhan K, Nanda S, Sharma A (2021). Platelet-rich fibrin, preparation and use in dermatology. Indian Dermatol Online J.

[15] Loesch MM, Somani AK, Kingsley MM, Travers JB, Spandau DF (2014). Skin resurfacing procedures: new and emerging options. Clin Cosmet Investig Dermatol.

[16] Asif M, Kanodia S, Singh K (2016). Combined autologous platelet-rich plasma with microneedling verses microneedling with distilled water in the treatment of atrophic acne scars: a concurrent split-face study. J Cosmet Dermatol.

[17] Fabbrocini G, De Vita V, Pastore F, Panariello L, Fardella N, Sepulveres R, D'Agostino E, Cameli N, Tosti A (2011). Combined use of skin needling and platelet-rich plasma in acne scarring treatment. Cosmet Dermatol.

[18] Pavlovic V, Ciric M, Jovanovic V, Stojanovic P (2016). Platelet rich plasma: a short overview of certain bioactive components. Open Med.

[19] Nofal E, Helmy A, Nofal A, Alakad R, Nasr M (2014). Platelet-rich plasma versus CROSS technique with 100% trichloroacetic acid versus combined skin needling and platelet rich plasma in the treatment of atrophic acne scars: a comparative study. Dermatol Surg.

[20] Tantari SH, Murlistyarini S (2016). Combination treatment of skin needling, platelet-rich plasma and glycolic acid 70% chemical peeling for atrophic acne scars in Fitzpatrick’s skin type IV–VI. J Clin Exp Dermatol Res.

[21] Wang X, Yang Y, Zhang Y, Miron RJ (2019). Fluid platelet-rich fibrin stimulates greater dermal skin fibroblast cell migration, proliferation, and collagen synthesis when compared to platelet-rich plasma. J Cosmet Dermatol.

[22] Shashank B, Bhushan M (2021). Injectable Platelet-Rich Fibrin (PRF): The newest biomaterial and its use in various dermatological conditions in our practice: A case series. J Cosmet Dermatol.

